# Effect of PEEP on breath sound power spectra in experimental lung injury

**DOI:** 10.1186/s40635-014-0025-y

**Published:** 2014-10-16

**Authors:** Jukka Räsänen, Michael E Nemergut, Noam Gavriely

**Affiliations:** Department of Anesthesiology, H. Lee Moffitt Cancer Center, 12902 USF Magnolia Drive, Tampa, FL 33612-9416 USA; Departments of Anesthesiology and Pediatrics, Mayo Clinic, 200 First Street SW, Rochester, MN 55905-0001 USA; Technion - Israel Institute of Technology, Rappaport Faculty of Medicine, 16 Palyam Ave., Haifa, 31096 Israel

**Keywords:** Acute lung injury, Respiratory sounds, Acoustics, Spectrum analysis

## Abstract

**Background:**

Acute lung injury (ALI) is known to be associated with the emergence of inspiratory crackles and enhanced transmission of artificial sounds from the airway opening to the chest wall. Recently, we described the effect of ALI on the basic flow-induced breath sounds, separated from the crackles. In this study, we investigated the effects of positive end-expiratory pressure (PEEP) on these noncrackling basic lung sounds augmented during ALI.

**Methods:**

Lung sounds were recorded in six anesthetized, intubated, and mechanically ventilated pigs at three locations bilaterally on the chest wall. Recordings were obtained before and after induction of lung injury with oleic acid and during application of incremental positive end-expiratory pressure.

**Results:**

Oleic acid injections caused severe pulmonary edema predominately in the dependent-lung regions. Inspiratory spectral power of breath sounds increased in all lung regions over a frequency band from 150 to 1,200 Hz, with further power augmentation in dependent-lung areas at higher frequencies. Incremental positive end-expiratory pressure reversed the spectral power augmentation seen with ALI, reducing it to pre-injury levels with PEEP of 10 and 15 cmH_2_O in all lung regions at all frequencies. The application of positive end-expiratory pressure to normal lungs attenuated spectral power slightly and only over a band from 150 to 1,200 Hz.

**Conclusions:**

We confirm a gravity-related spectral amplitude increase of basic flow-induced breath sounds recorded over lung regions affected by permeability-type pulmonary edema and show that such changes are reversible by alveolar recruitment with PEEP*.*

**Electronic supplementary material:**

The online version of this article (doi:10.1186/s40635-014-0025-y) contains supplementary material, which is available to authorized users.

## Background

The loss of alveolar capillary membrane integrity associated with acute lung injury induces accumulation of fluid in the extravascular space, gravity-dependent loss of gas volume, and profound alterations in the acoustic properties of the respiratory system [1-5]. The best known acoustic abnormality developing in injured lungs is the appearance of adventitious sounds such as crackles, as small airways begin to undergo cyclic collapse and reopening during breathing. However, the increased density of injured lung parenchyma also increases the propagation speed of natural or external sound signals and enhances sound energy transfer from inside the thorax to the surface of the chest, as the acoustic impedance difference between the parenchyma and the chest wall decreases [4,5]. While qualitative assessment of crackles is possible with a stethoscope, the spectral content, velocity, and power of natural and external sounds are amenable to computer-aided analysis making acoustic monitoring of lung injury potentially clinically feasible in a continuous, noninvasive fashion.

Studies evaluating the transmission of external, artificial sound introduced into the airway opening of injured lungs demonstrate that the application of positive end-expiratory pressure (PEEP) reverses the injury-induced increase in acoustic wave propagation speed and power transmission [1,3,5]. Using natural breath sounds as the interrogating signal, attractive for its simplicity, has yielded similar results but is confounded by the appearance and disappearance of adventitious sounds such as crackles with injury and PEEP, as they are part of the inspiratory spectral content [4]. Since crackles are acoustic phenomena secondary to rapid opening of small airways and they only arise from lung areas that undergo cyclic opening and closure during the respiratory cycle, it is important to separate the information contained therein from that of the breath sound spectra themselves. We have discovered that in experimental lung injury there is a period in the early part of inspiration where adventitious sounds are absent and that spectral analysis of this crackle-free segment also reflects the magnitude of injury [6]. These early inspiratory spectral changes may provide a robust acoustic target for monitoring lung injury if they respond consistently to injury and therapy. We designed this study to investigate whether early inspiratory spectral characteristics of lung sounds in established lung injury would reliably reflect reaeration and deaeration of the lung on and off incremental PEEP.

## Methods

After approval by the Institutional Animal Care and Use Committee of Mayo Clinic (A363E01), eight healthy pigs weighing 35 to 45 kg and cared for according to the current guidelines for the care and use of laboratory animals were included in the study. The standard anesthetic management and instrumentation for this experimental model has been described previously [2].

Breath sounds were recorded using six PPG sensors (linear 50 to 2,000 Hz, response range 10 to 5,000 Hz, PPG-02, Technion, Haifa, Israel) placed on the chest wall. The six sensors were positioned midway between the apex and base of the lung bilaterally in three locations: 1) posteriorly 5 cm lateral from the spine, 2) anteriorly 5 cm lateral from the edge of the sternum, and 3) on the side, halfway between the other two locations. The sensors were secured to the shaved chest with a circumferential elastic strip such that contact force was sufficiently firm, but chest expansion was unimpeded. The signal from each sensor was amplified (Blue tube, PreSonus, Baton Rouge, LA, USA) and digitized (10,000 samples/s, 16-bit) into a portable computer using a 16-channel, 16-bit analog/digital converter board (PCI-6035E, National Instruments, Austin, TX, USA) inserted into a PCI expansion system (CB1F, Magma, San Diego, CA, USA). Each sound recording was 10 s in duration and contained three respiratory cycles of which two complete and artifact-free ones were selected for analysis. All sound measurements were made in duplicate.

At each phase of this experiment, after breath sounds were recorded, a broad-band sound signal was introduced into the airway during a 10-s respiratory hold for measurement of transfer function magnitude and phase and coherence of the external signal. The results of these measurements have been reported earlier [3,5].

### Experimental protocol

Thirty minutes after instrumentation, baseline respiratory volume and pressure recordings, blood gas sampling, hemodynamic measurements, and sound data collection were performed at PEEP of 0 cmH_2_O. The measurements were repeated after 15 min of ventilation with 10 cmH_2_O PEEP and again after 15 min with no PEEP. Acute lung injury was then induced with intravenous oleic acid, as described previously [2].

When the deterioration of oxygenation caused by the oleic acid injection reached a plateau, data collection was repeated after 15 min of ventilation with 0, 5, 10, and 15 cmH_2_O PEEP and concluded with two measurements, 15 min apart, after 15 min of ventilation with no PEEP. Thereafter, the animal was exsanguinated and a postmortem examination of the lungs was performed.

### Data analysis

Venous admixture and static respiratory system compliance were calculated from standard formulae. The sound recordings for each sensor and each phase of the study were analyzed using sound processing software (Adobe Audition CS6, Adobe, San Jose, CA, USA). Since the inspiratory adventitious sounds (crackles) are late in inspiration, the beginning of each inspiration was identified visually from a spectral display and a 150-ms segment was selected starting 50 ms into the inspiration as described previously [6]. Visual and auditory examinations of the segment were used to ascertain that no adventitious sounds were included in the selection. If such sounds were present, the length of the selection was reduced to 100 ms to exclude the adventitious sound. The selected segment was scanned, and an ensemble average of 256-point fast Fourier transformation (FFT) spectra was generated using a Blackmann-Harris window with 50% overlap (i.e., 12.3 ms). The FFT spectra of two inspirations were averaged at each frequency point to yield the final power spectrum for that recording. The spectra from the duplicate recordings at each phase of the study were then averaged. As such, each averaged spectrum consisted of data from approximately 40 FFT segments representing four breaths. A band of 150 to 3,000 Hz of the frequency spectrum was isolated for quantitative analysis and divided into sub-bands as needed in a Microsoft Excel spreadsheet. The mean spectral power within each band was calculated as the mean of the FFT values belonging to the band. Data from sensors overlying the corresponding regions of the two sides of the chest, nondependent lung, mid lung and dependent lung, were combined for analysis of the effect of gravity on the power spectra.

To investigate in detail the changes caused by PEEP and injury on spectral power, we subtracted the baseline pre-injury spectral values at each frequency point from the values obtained at other phases of the study. The baseline recordings before the application of 10 cmH_2_O PEEP on normal lungs and the one recorded immediately prior to injection of oleic acid were used to assess the effects of PEEP on normal and injured lungs, respectively. Data from the last measurement, 30 min after discontinuation of PEEP, did not yield additional information and are therefore not included.

### Statistical methods

The results are presented as mean ± standard deviation (SD) of the animals that produced analyzable data. The statistical significance of the observed changes was evaluated using the Wilcoxon signed rank test or Friedman's one-way, repeated-measures analysis of variance applied to the three lung regions separately. The effects of PEEP before and after injury were analyzed independently. If a statistically significant overall change was found in the analysis of variance, individual differences between the phases of the study were isolated using Dunn's multiple comparison test (GraphPad Prism 6, GraphPad Software Inc., La Jolla, CA, USA). Pairwise testing of the injury effect was limited to comparing the injured state at 0 cm PEEP with all other PEEP phases. Pearson's linear correlation coefficients were calculated to compare the power spectrum changes with physiological variables reflecting oxygenation and lung mechanics. Results with *p* values less than 5% were considered statistically significant.

## Results

### Physiologic measurements

In six of the eight studied animals, lung sound recordings from all sensors were of sufficient quality to be analyzed. Data from two animals were excluded because of a missing recording at PEEP 15 cmH_2_O in one and a poor signal in one sensor in another. The application of 10 cm PEEP to uninjured lungs caused expected decreases in blood pressure and cardiac output (Table 1). Venous admixture also decreased significantly for the duration of ventilation with PEEP but without a statistically significant improvement in oxygenation.

**Table 1:** **The pre-injury effect of 10 cmH**_**2**_ **O PEEP on variables reflecting cardiopulmonary function and breath sound spectral power in six anesthetized, mechanically ventilated uninjured pigs**

	**PEEP 0**	**PEEP 10**	**PEEP 0**	***p*** **value**
Heart rate (bpm)	86 ± 16	84 ± 12	81 ± 13	NS
Mean blood pressure (mmHg)	88 ± 13	74 ± 11	93 ± 11	<0.01
Cardiac output (L/min)	4.0 ± 0.4	2.6 ± 0.7	3.9 ± 0.6	<0.01
P_a_O_2_ (mmHg)	92 ± 18	102 ± 20	97 ± 16	NS
P_a_CO_2_ (mmHg)	37 ± 2	37 ± 3	39 ± 2	NS
pHa	7.44 ± 0.01	7.45 ± 0.03	7.42 ± 0.02	NS
SaO_2_ (%)	94 ± 3	95 ± 3	94 ± 4	NS
Venous admixture (%)	19 ± 13	10 ± 12	13 ± 14	<0.05
C_st_ (ml/cmH_2_O)	30 ± 6	26 ± 4	33 ± 4	NS
Average spectral power (dB)	−65.2 ± 7.8	−68.3 ± 4.8	−67.6 ± 3.9	NS
Spectral power 150 to 1,200 Hz (dB)	−60.1 ± 7.1	−64.2 ± 4.7	−62.6 ± 3.2	<0.05
Spectral power 1,200 to 3,000 Hz (dB)	−67.8 ± 8.2	−70.4 ± 4.9	−70.3 ± 4.4	NS

C_st_, static respiratory system compliance; NS, not statistically significant. Physiologic data published previously [3,5]. Values are mean ± SD. *p* values refer to overall effect of PEEP (Friedman's analysis of variance).

Induction of lung injury was accompanied by expected tachycardia, systemic hypotension, and pulmonary hypertension, while cardiac output remained unchanged (Table 2). A 4-fold increase in venous admixture required oxygen supplementation to prevent profound hypoxemia. The static respiratory system compliance decreased to 56% of its baseline value.

**Table 2:** **The effect of oleic acid-induced lung injury, and incremental application of PEEP on variables reflecting cardiopulmonary function in six anesthetized, mechanically ventilated pigs**

	**No ALI**	**ALI**	**ALI**	**ALI**	**ALI**	**ALI**	***p*** **value**
	**PEEP 0**	**PEEP 0**	**PEEP 5**	**PEEP 10**	**PEEP 15**	**PEEP 0**	
Heart rate (bpm)	81 ± 14	92 ± 12	91 ± 17	94 ± 18	100 ± 14	94 ± 8	NS
Mean blood pressure (mmHg)	93 ± 11	68 ± 4	66 ± 4	67 ± 4	76 ± 6	78 ± 9	<0.001
Core temperature (°C)	35.7 ± 0.5	35.5 ± 0.6	35.6 ± 0.6	35.5 ± 0.6	35.5 ± 0.6	35.5 ± 0.6	NS
Cardiac output (L/min)	3.9 ± 0.6	4.0 ± 0.6	3.7 ± 0.6	3.2 ± 0.5	3.0 ± 0.4	4.1 ± 0.6	<0.001
P_a_O_2_ (mmHg)	97 ± 17	82 ± 12	119 ± 33	174 ± 32	237 ± 33	76 ± 8	<0.001
P_a_CO_2_(mmHg)	39 ± 2	51 ± 6	47 ± 4	45 ± 4	44 ± 5	50 ± 6	<0.001
pH_a_	7.42 ± 0.02	7.33 ± 0.03	7.35 ± 0.04	7.37 ± 0.03	7.37 ± 0.04	7.34 ± 0.03	<0.001
S_a_O_2_	94 ± 4	91 ± 4	95 ± 2	97 ± 1	98 ± 0	90 ± 4	<0.001
Ventilator rate (cpm)	17 ± 2	20 ± 2	20 ± 2	20 ± 2	20 ± 2	21 ± 2	<0.001
Tidal volume (ml)	377 ± 25	357 ± 29	370 ± 15	370 ± 15	365 ± 21	365 ± 23	NS
F_I_O_2_	0.22 ± 0	0.52 ± 0.22	0.52 ± 0.22	0.52 ± 0.20	0.53 ± 0.21	0.62 ± 0.26	NT
Venous admixture (%)	13 ± 14	57 ± 13	45 ± 18	32 ± 19	16 ± 18	65 ± 11	<0.001
C_st_ (ml/cmH_2_O)	33 ± 5	16 ± 3	19 ± 3	22 ± 3	23 ± 2	18 ± 3	<0.001

ALI, acute lung injury; C_st_, static respiratory system compliance; NS, not statistically significant; NT, variable not statistically tested. Physiologic data published previously [3,5]. Values are mean ± SD. *p* values refer to the overall effect of PEEP (Friedman's analysis of variance).

The incremental application of 5 to 15 cmH_2_O PEEP decreased cardiac output but improved respiratory system compliance and returned venous admixture to pre-injury levels. A reversal of these effects was observed within 15 min of discontinuing PEEP. In all cases, examination of the lungs at the end of the study revealed consolidation of the dependent part of both lungs over a fifth to a third of their vertical height.

### Sound measurements

The application of PEEP to normal lungs was not associated with statistically significant change in breath sound spectral power when measured over the entire frequency band from 150 to 3,000 Hz (Table 1). However, examination of the change in spectral power with application of PEEP revealed a small frequency-dependent attenuation of power in all lung regions. This attenuation was found to be statistically significant at frequencies from 150 to 1,200 Hz (*p* < 0.05), but not at higher frequencies.

**Figure 1:**
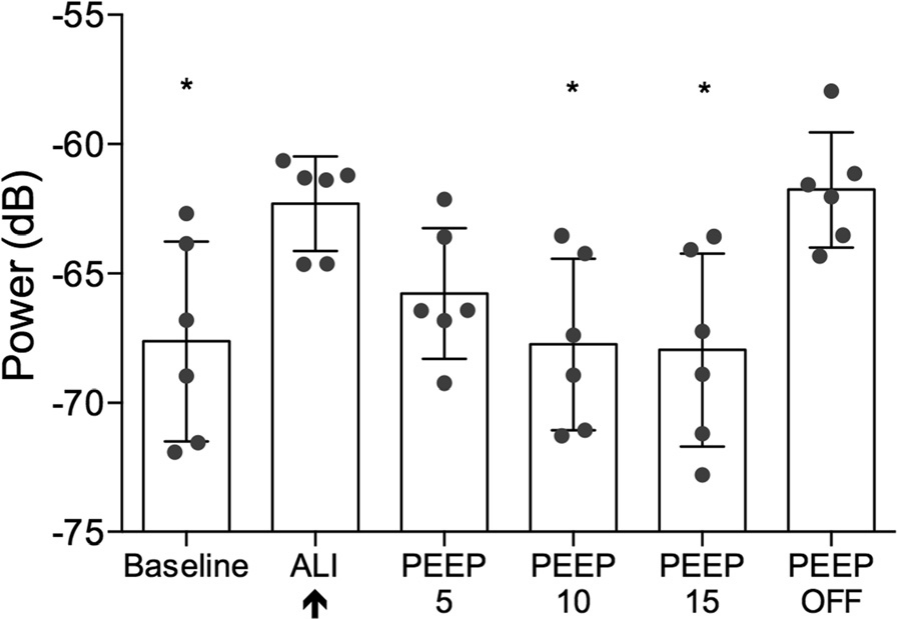
Changes in average (± SD) and individual (dots) spectral power with lung injury (arrow) and PEEP (*: p < 0.05).

**Figure 2:**
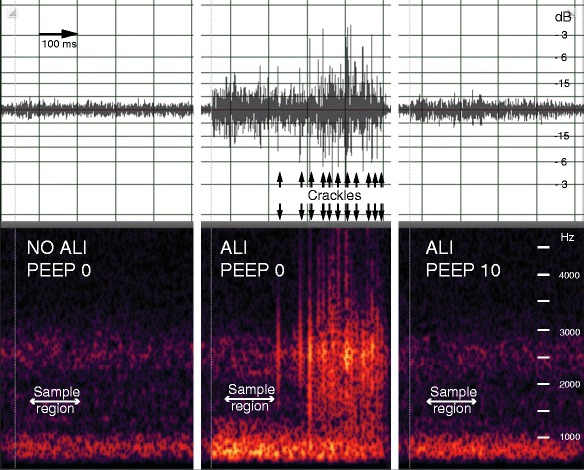
**Single inspiratory waveform (top) and spectral (bottom) displays from the right midlung of a representative animal showing sample regions and late inspiratory crackles.** The respective audio recordings are available (Additional files 1, 2, 3).

Oleic acid lung injury increased the average spectral power of breath sounds (*p* < 0.05) when calculated over the entire measurement band across all six sensors (Figure 1). Incremental application of PEEP to injured lungs attenuated this increase at all levels. The effect was partial with 5 cmH_2_O of PEEP but already maximal at 10 cmH_2_O PEEP and returned the spectral power to baseline values. Application of 15 cmH_2_O of PEEP did not decrease the spectral power below pre-injury levels. Upon discontinuation of PEEP, the acoustic power spectra returned to the elevated pre-PEEP levels. Recordings of single inspirations from a right mid-lung sensor of a representative animal before injury, after injury without PEEP, and after injury with 10 cmH_2_O PEEP in both waveform and spectral view are shown in Figure 2. The respective audio files are available for listening (Additional files 1, 2, 3).

**Figure 3:**
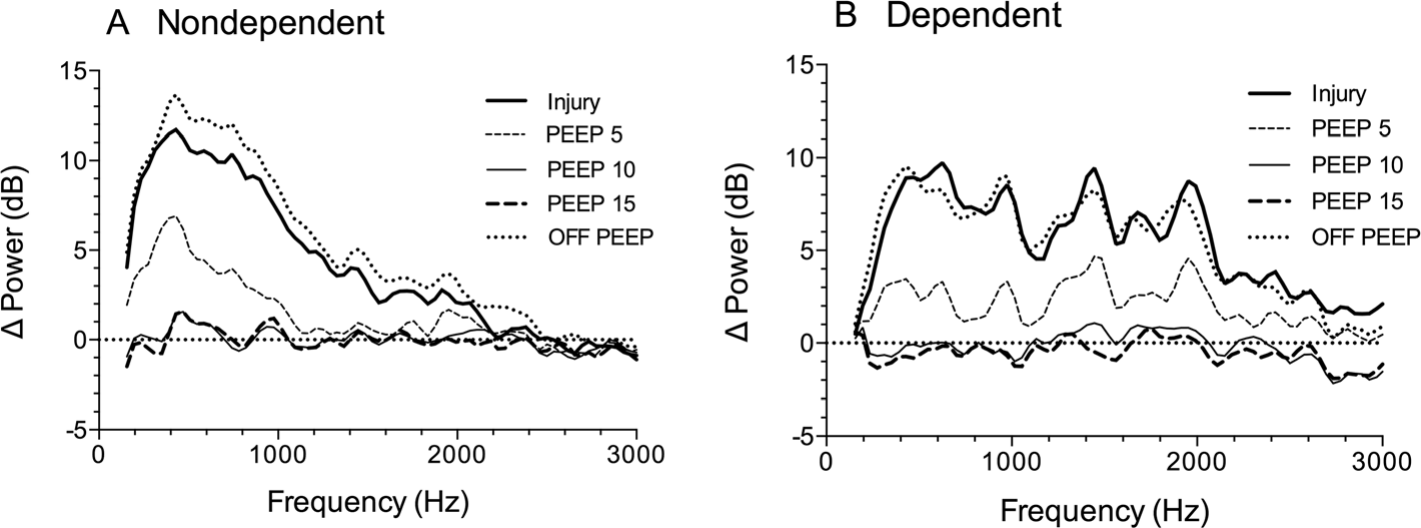
**Effect of sensor location over nondependent vs. dependent lung on spectral changes from lung injury and PEEP.**

**Figure 4:**
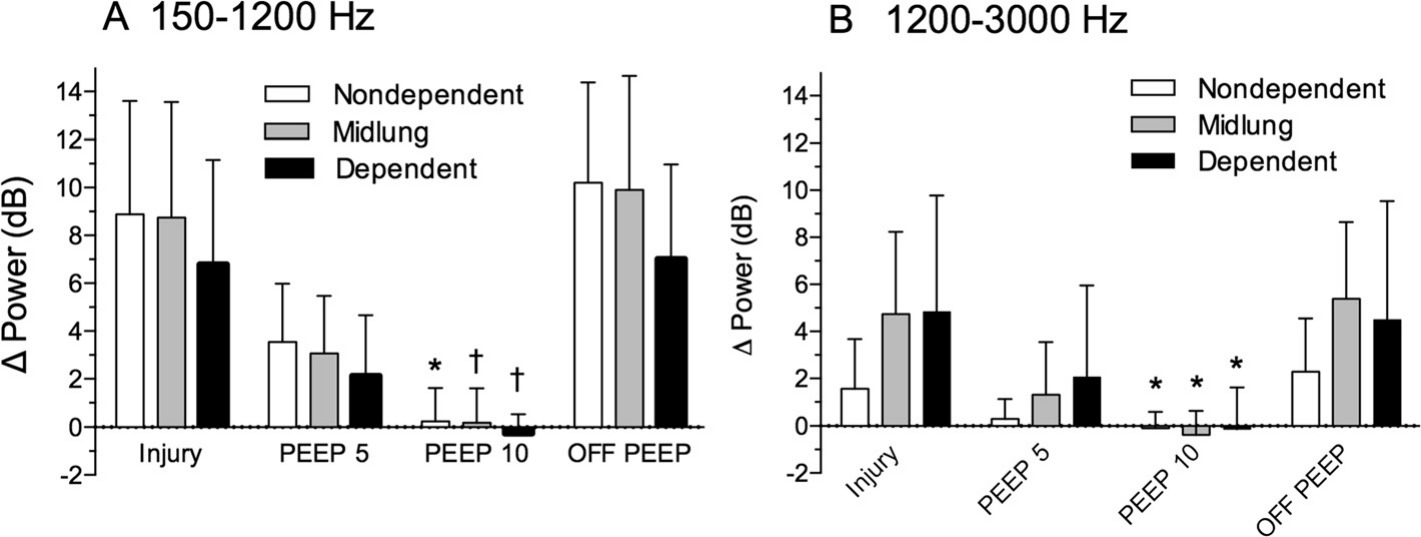
Spectral changes (mean±SD) broken down by sensor location at low (panel A) and high (panel B) frequency ranges (*: p < 0.05, †: p < 0.01 compared to the injured state before PEEP).

The spectral power response to injury depended upon the gravitational region of the lung (Figure 3), a finding that has been reported previously [6]. All sensors showed an equivalent increase in power over a frequency band from 150 to 1,200 Hz. At higher frequencies, the increase was minimal in nondependent-lung regions (Figure 3A); in the mid- and dependent-lung regions, the effect was substantially larger (Figure 3B). Regardless of sensor location or frequency band, however, an incremental and statistically significant decrease in spectral power was observed as PEEP was applied (Figures 3 and 4). Five cmH_2_O PEEP decreased the spectral power increment to approximately half of its injury-induced value, while 10 cmH_2_O PEEP abolished the effect of injury on the acoustic power spectra. Increasing the PEEP to 15 cmH_2_O did not produce further reduction.

**Figure 5:**
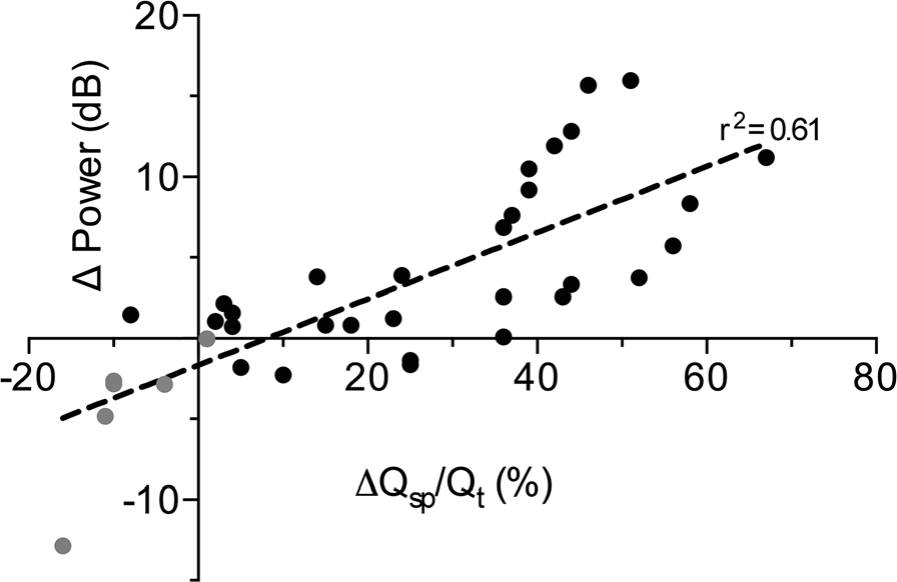
**All data points of venous admixture plotted against concurrent change in spectral power over nondependent lung before (grey dots) and after (black dots) lung injury.**

The average within-subject linear correlation coefficients (*r*^2^) between change in average spectral power and change in venous admixture and static compliance from their baseline values were 0.76 ± 0.16 and 0.60 ± 0.23, respectively. Linear correlation of the change in venous admixture plotted against the corresponding change in the low-frequency (150 to 1,200 Hz) spectral power had an explained variation (*r*^2^) of 0.61 calculated from all data points of all subjects (Figure 5).

## Discussion

Preventing cyclic collapse and reopening of alveoli during tidal ventilation is an established principle to minimize the risk of ventilator-induced lung injury in critically ill patients [7] and, recently, even in patients with normal lungs mechanically ventilated in the operating room [8]. The absence of simple, noninvasive and continuous methods to evaluate the aeration of lungs has renewed interest in the analysis of the acoustic characteristics of the respiratory system during mechanical ventilation. Both over- and under-inflation of the lungs induce changes in the transmission of both externally introduced and breathing-induced lung sounds [3,9,10]. As such, monitoring the acoustic properties of the lung could potentially be used to identify areas of injury, follow lung inflation, and optimize ventilator settings during the recruitment of collapsed lung parenchyma.

Even if inspiratory flow rate and tidal volume are held constant, several factors influence the acoustic power of the breath sound signal reaching a chest surface sensor when lung aeration changes. Collapse of lung units will direct ventilation to areas that are still open and enhance sound generation by way of increased regional inspiratory flow rate and Reynold's number. In the collapsed region, the smaller acoustic impedance mismatch at the boundary between lung parenchyma and chest wall allows for enhanced transfer of sound energy to the chest surface. Over inflation of the lungs increases airway diameter and alters the propagation characteristics of the acoustic wave front. By increasing the acoustic impedance gap between the lung and the chest wall, hyperinflation attenuates acoustic energy transfer to the surface sensor. Depending on the nature of the lung disorder, adventitious sounds may be generated, with concomitant alterations in the composition of the sound signal.

Prior research has shown that an externally introduced sound signal can be used to monitor progress and regress of lung injury by calculating its transfer function magnitude and wave speed as it traverses from the airways onto the chest surface [3,5]. The spectral power of flow (turbulence)-induced acoustic signals, breath sounds, and disease-related adventitious sounds (crackles) taken together, will respond in a similar fashion [4]. However, crackles represent opening of small airways in areas of the lung subject to expiratory closure, while sound spectra themselves appear to reflect aeration of the parenchyma underneath the sensor. Since these two variables represent different physiological characteristics, they should be analyzed separately, if possible. Use of an externally introduced sound signal avoids the variability and quantification problems from adventitious sounds but requires complex equipment. We sought to determine whether a ‘clean’ early inspiratory power spectrum of natural breath sounds could be found consistently and used to track injury and recovery.

The results of this study confirm our and others' findings that acute permeability-type lung injury increases the acoustic power of breath sounds detected at the surface of the chest over a wide frequency range [3,4,6,10]. In a previous study, we monitored this increase, excluding adventitious sounds, for the first hour of developing injury in prone and supine animals and found an increase in breath sound power within a frequency band from 150 to 800 Hz regardless of whether the sensor was overlying a nondependent, aerated lung or a dependent, deaerated lung [6]. At higher frequencies (800 to 3,000 Hz), this increase was observed over dependent-lung areas only and followed the gravitational position of the sensor regardless of the prone or supine positions. In the present study, we confirmed this gravity dependency of the power spectrum frequency distribution in established injury and further discovered that at all sensor locations the injury-induced increase in spectral power is reversed if the lung parenchyma is expanded with PEEP.

When injured lungs are ventilated without positive expiratory airway pressure, some areas of the lung parenchyma are likely to remain deaerated throughout the ventilatory cycle, others remain open, and yet others open as lung volume increases during inspiration and close in expiration. The reopening of closed airways will generate discontinuous adventitious lung sounds starting late in inspiration. If PEEP is able to maintain the airways leading to these areas open, the presence of such sounds should vary with the level of PEEP. Thus lung injury and the application of PEEP to injured lungs would be expected to change the lung sound power spectrum when studied inclusive of crackles, as has indeed been reported [4]. In this study, we sampled the breath sounds at early inspiration before the onset of crackles to exclude all adventitious sounds. The changes we observed therefore are not the result of appearance or disappearance of crackles.

No single factor can explain the increase in spectral power over both aerated and deaerated lung. Since we held the inspiratory flow rate and tidal volume constant and excluded adventitious sounds, we suggest that the observed changes reflect a combined effect of increased sound transmission and redistribution of inspiratory gas flow. Areas of lung consolidation during oleic acid-induced lung injury provide less acoustic impedance mismatch to the transfer of sound energy to the chest surface and enhance recorded spectral power even when air-to-tissue ratio in these areas is reduced (increased lung density). Concurrently, as ventilation is redistributed from dependent to nondependent lung, breath sound power is enhanced in nondependent, aerated regions as well. While all sensors recorded an increase in power at 150 to 1,200 Hz, the fact that the only sensors that revealed increased spectral power at 1,200 to 3,000 Hz were overlying dependent-lung areas suggests that enhanced transmission is the primary mechanism by which spectral power is increased at these higher frequencies. This corresponds to the increased transfer function magnitude in the higher-frequency range observed previously using an external signal [2,3].

The reaeration of lung with PEEP would be expected to rebalance regional ventilation, reduce lung density, and eliminate the enhanced transmission at the chest wall boundary and thus eventually restore baseline power spectra. While this occurred gradually with incremental increase in PEEP, the spectral changes were essentially complete with only 10 cmH_2_O. Increasing the PEEP to 15 cmH_2_O did not further change the power spectra or respiratory system compliance despite improving oxygenation. It is possible that the power spectral changes depend on relatively large changes in aeration, which in our subjects was mostly complete at PEEP of 10 cmH_2_O. More modest recovery in the ventilation perfusion ratio may still continue to improve oxygenation at higher PEEP levels. A good correlation between change in spectral power and venous admixture is further proof that these variables both reflect the degree of lung aeration during injury and recruitment (Figure 5). Hyperinflation-induced attenuation of spectral power would not be expected in injured lungs at 15 cmH_2_O of PEEP because even in normal lungs PEEP levels of 10 cmH_2_O or higher are required to produce this effect [9].

## Conclusions

Our results confirm that changes in the breath sound power spectrum occur consistently during the development of permeability-type acute lung injury when determined away from adventitious sounds and that these changes contain information that differentiates between more and less severely affected lung regions. As such, breath sound spectra free from interference by adventitious sounds could prove useful in the clinical management of patients with lung injury. Restoration of lung volume with PEEP reverses the spectral changes entirely, suggesting that the spectral power abnormalities depend primarily on the severity of regional gas volume loss in the pulmonary parenchyma and could potentially be used to optimize the level of PEEP. It is conceivable that a comprehensive acoustic lung assessment tool could be constructed, with concurrent assessment of spectral changes, sound transmission magnitude, acoustic wavefront velocity, and the number and location of crackles. Such a tool may provide a valuable, non-invasive means to optimize ventilatory support for patients with acute lung injury.

## Additional files

Additional file 1:
**Recording of breath sounds from a sensor located over the right mid-lung region of a representative animal during mechanical ventilation without PEEP before induction of lung injury.**
Additional file 2:
**Recording of breath sounds from a sensor located over the right mid-lung region of a representative animal with oleic acid-induced acute lung injury during mechanical ventilation without PEEP.**
Additional file 3:
**Recording of breath sounds from a sensor located over the right mid-lung region of a representative animal with oleic acid-induced acute lung injury during mechanical ventilation with 10 cmH2O PEEP.**
